# Is acupuncture effective and safe for prophylaxis of vestibular migraine? A protocol for systematic review and meta analysis

**DOI:** 10.1097/MD.0000000000023533

**Published:** 2020-12-18

**Authors:** Tianye Hu, Aijun Zhang, Bin Jiang, Fengfei Shen, Jin Hu

**Affiliations:** a Department of Traditional Chinese Medicine and Acupuncture; b Department of Neurology, The First Affiliated Hospital of Jiaxing University, Jiaxing 314001, Zhejiang Province, China.

**Keywords:** acupuncture, efficacy, meta-analysis, prophylaxis, vestibular migraine

## Abstract

**Background:**

Increasing studies indicate that acupuncture can be used for treating vestibular migraine (VM), but current evidence remains inconclusive. Thus, this protocol aims to evaluate the evidence regarding the efficacy and safety of acupuncture for VM prophylaxis by conducting a systematic review and meta-analysis.

**Methods:**

Studies will be retrieved by searching electronic databases from their inception to December 2020, including EMBASE, PubMed, Web of Science, Cochrane Library, Chinese National Knowledge Infrastructure (CNKI), Chinese BioMedical Literature Database (CBM), and Chinese Science and Technology Periodical Database (VIP). Eligible randomized controlled trials involving acupuncture for VM prophylaxis will be included. Study screening, data collection, and assessment for risk of bias will be executed by 2 independent reviewers. Meta-analyses will be conducted, followed by subgroup analysis if significant heterogeneity is detected. Sensitivity analysis and summary of the strength of the evidence will also be performed.

**Results:**

The results of the present systematic review and meta-analysis will verify the efficacy and safety of acupuncture for VM prophylaxis.

**Conclusion:**

This review will determine the efficacy and safety of acupuncture on VM prophylaxis. The findings are expected to verified whether acupuncture can be an alternative treatment for VM prophylaxis.

**Ethics and dissemination:**

Given that a systematic review and meta-analysis will not involve private information of individuals, ethical approval is not required. Relevant results and findings will be submitted to an academic journal for peer reviews.

**PROSPERO registration number:**

CRD42020202588.

## 1 Introduction

Vestibular migraine (VM) is one of the most common causes for vestibular diseases, which ranks the second place following benign paroxysmal positional vertigo (BPPV).^[1]^ Its clinical features include recurrent episodic vestibular symptoms, a previous or current migraine history, and migrainous symptoms (e.g., migrainous headache, phonophobia, photophobia, visual or other neurologic auras).^[1]^ Its major symptoms of vertigo and migraine can coexist in 3 ways, in which vertigo can attack before, or after, or coincident with migraine.^[2]^ VM can occur at any age, but the most common age category is between 30 and 40 years. For gender differences, VM occurs about 1.5 to 5 times more often in females than in males.^[3,4]^ Epidemiological surveys revealed that its 1-year prevalence is 2.7% among adults in the USA.^[5]^ The lifetime prevalence of VM is about 1% and a 1-year prevalence of VM is about 0.9% in general population.^[6]^ Moreover, it accounts for about 9% of patients in migraine clinics,^[1]^ 11% of patients in dizziness clinics,^[7]^ and 4.2% to 29.3% of patients in otolaryngology clinics.^[8,9]^ Nevertheless, the accurate diagnostic of VM is low. A recent study found that the referring doctors had suspected only 1.8% of the young patients to have VM in a tertiary vertigo center, whereas a diagnosis was made in 20.2%.^[10]^ Common comorbidities of VM include Meniere disease and emotional disorders, especially anxiety and depression, which have brought a huge global burden of disease.^[11–13]^ Nevertheless, the pathogenesis of VM remains poorly understood and needs further investigation.

At present, the treatment of VM mainly targets at acute stage and prophylaxis. Pharmacological treatments for acute VM mainly choose triptans and pharmacological treatments for VM prophylaxis include western medication for preventing migraine, such as metoprolol, propranolol, valproic acid, topiramate, and amitriptyline. However, pharmacological treatments are often associated with various side effects, such as dizziness, paraesthesia, dyspepsia, nausea, dry mouth, chest discomfort, and somnolence.^[14]^ Frequent VM attack can seriously affect patients’ daily life and work, thus, prophylaxis of VM is of great significance for patients. There is a consensus among experts that prophylaxis treatment is vital for VM patients if the frequency of vertigo/migraine attacks is at least 3 times per month in the last 3 months, or patients have poor response to treatment in the acute phase of VM.^[15]^

As an important component of complementary and alternative medicine, acupuncture can promote recovery of health by inserting needles and stimulating specific points on the body. Given to its role as an effective and safe nonpharmaceutical therapy for treating a wide range of diseases, acupuncture is gaining acceptance all over the world. Acupuncture is widely used in treating migraine and vertigo/dizziness,^[16,17]^ which are major symptoms of VM. Meanwhile, it can also improve emotional disorders,^[18]^ which are common comorbidities in VM. Thus, acupuncture might be a favorable therapy for treating VM. In recent years, there are increasing clinical trials investigating the efficacy and safety of acupuncture for VM prophylaxis, but current evidence is inconclusive.^[19]^ Therefore, this will be a protocol for the first systematic review and meta-analysis that aims to ascertain the evidence for efficacy and safety of acupuncture on VM prophylaxis.

## 2 Methods

### 2.1 Study design and registration

This protocol for a systematic review and meta-analysis is conducted in accordance with the Preferred Reporting Items for Systematic Reviews and Meta-analyses Protocols (PRISMA-P)^[20]^ to report the findings.

This PRISMA-based systematic review and meta-analysis has been registered in PROSPERO with the registration number CRD42020202588.

### 2.2 Eligibility criteria for including studies in this review

#### 2.2.1 Types of studies

We will include studies of randomized controlled trials (RCTs) involving acupuncture for VM prophylaxis. Studies in nonrandomized or uncontrolled designs will be excluded. Language restrictions will not be imposed.

#### 2.2.2 Types of participants

Participants with a confirmed diagnosis of VM based on internationally recognized diagnostic criteria (e.g., the criteria proposed by the collaboration of Barany Society and the International Headache Society in 2012) will be included. Meanwhile, patients need prophylaxis treatment when vertigo/migraine attacks at least 3 times per month in the last 3 months, or vertigo/migraine days are at least 4 days per month, or patients have poor response to treatment in the acute phase of VM. All patients with age ≥18 years, male or female.

Participants of the following conditions will be excluded: Patients do not meet the diagnostic criteria and age criteria; and vertigo and headache are caused by other diseases, such as BBPV.

#### 2.2.3 Intervention

##### 2.2.3.1 Modalities of experimental interventions

The modalities of experimental interventions include acupuncture therapy alone, and acupuncture combined with another widely recognized active therapy (e.g., pharmacologic treatment for VM prophylaxis). Acupuncture therapy is defined as any acupuncture modality, such as manual acupuncture, electroacupuncture, auricular acupuncture, scalp acupuncture, warming-needle moxibustion, and so on.

##### 2.2.3.2 Modalities of control interventions

The modalities of control interventions include widely recognized active therapies for prophylaxis of VM, such as preventive drugs; no treatment or waiting list; and placebo controls: such as sham acupuncture, placebo drugs, sham interventions.

#### 2.2.4 Outcome measures

##### 2.2.4.1 Types of primary outcomes

Primary outcomes consist of vertigo/migraine episodes: including vertigo/migraine days and vertigo/migraine frequency per month; intensity of vertigo/migraine measured by acknowledged scales, such as visual analogue scale (VAS); and response rate.

##### 2.2.4.2 Types of secondary outcomes

Secondary outcome measures include vertigo-related assessment scale, such as the Dizziness Handicap Inventory (DHI) scale; anxiety level assessed by valid questionnaires, such as the Generalized Anxiety Disorder-7 (GAD-7) scale and the Hamilton Anxiety Scale (HAMA); depression level measured by valid questionnaires, such as the Patient Health Questionnaire (PHQ-9) scale and the Hamilton Depression Scale (HAMD);patients’ quality of life measured by acknowledged scales, such as the 36-Item Short Form Health Survey (SF-36); and adverse events.

### 2.3 Data sources and retrieval strategy

Studies will be retrieved by searching the following electronic databases from their inception to December 2020, which include PubMed, Web of Science, EMBASE, Cochrane Library, Chinese National Knowledge Infrastructure (CNKI), Chinese BioMedical Literature Database (CBM), and Chinese Science and Technology Periodical Database (VIP). We will also scan references of all eligible literature to identify additional publications. The search strategies are based on a combination of subject headings [e.g., Medical Subject Headings (MeSH) in PubMed] and the following search terms: acupuncture, needling, manual acupuncture, electroacupuncture, auricular acupuncture, warming-needle moxibustion, vestibular migraine, migraine-associated dizziness, migraine-associated vertigo, vertiginous migraine, hemicrania, cephalalgia, cephalagra, migraine and randomized controlled. Corresponding terms in Chinese will be searched in Chinese databases. The example of search strategy for PubMed is displayed in Table 1.

**Table 1:** Search strategy in PubMed.

No.	Search items
#1	Randomized controlled trial [pt]
#2	Controlled clinical trial [pt]
#3	Randomized OR Randomised [Title/Abstract]
#4	Clinical trials [MeSH]
#5	Randomly [Title/Abstract]
#6	Trial [Title/Abstract]
#7	#1 OR #2 OR #3 OR #4 OR #5 OR #6
#8	Humans [MeSH]
#9	#7 AND #8
#10	vestibular migraine [MeSH]
#11	vestibular migraine OR migraine-associated dizziness OR migraine-associated vertigo OR vertiginous migraine [Title/Abstract]
#12	hemicrania OR cephalalgia OR cephalagra OR migraine [Title/Abstract]
#13	#10 OR #11 OR #12
#14	Acupuncture therapy OR electroacupuncture therapy [MeSH]
#15	(Acupuncture OR electroacupuncture OR electro-acupuncture OR manual acupuncture OR auricular acupuncture OR ear acupuncture OR warm needling OR warming-needle moxibustion) [Title/Abstract]
#16	#14 OR #15
#17	#9 AND #13 AND #16

### 2.4 Data collection and statistical analysis

#### 2.4.1 Study selection and data extraction

**Figure 1:**
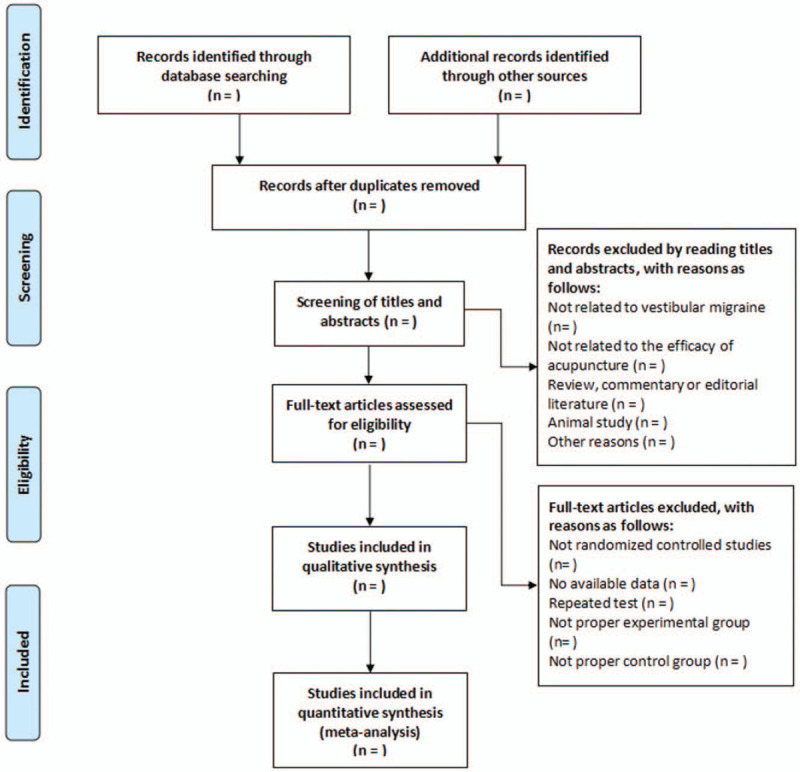
Flow diagram of study selection process.

Two independent reviewers (TYH and JH) will screen papers to include eligible RCTs by reading titles, abstracts of studies according to the inclusion/exclusion criteria. The full text of potentially qualified studies will be reviewed to confirm eligibility. The study screening process in a flow chart is displayed in Figure 1. When all included studies are determined, the same 2 reviewers will perform data extraction in terms of study design, characteristics of study population, intervention types, control types, treatment courses, treatment sessions, primary and secondary outcomes, and so on. Any disagreements during data extraction will be settled by consulting a third reviewer (JH). Regarding data of continuous outcomes, they will extract the mean difference (MD), standard deviation (SD) in each group, and the total number of participants. Regarding dichotomous outcomes, they will extract the number of responders as well as the total number of patients in each group.

#### 2.4.2 Assessment for risk of bias

Two reviewers (TYH and FFS) will perform the assessment of risk of bias independently based on the Cochrane risk-of-bias tool. In details, risk of bias of a included study will be graded into 3 categories as either: “unclear,” “low,” or “high” risk for 7 domains as follows: random sequence generation, allocation concealment, blinding of participants and personnel, blinding of outcome assessment, incomplete data, selective outcome reporting, and other bias.^[21]^ If there is controversy during assessment for risk of bias, it will be arbitrated by a third reviewer (JH).

#### 2.4.3 Measures of effect sizes

Effect sizes will be measured by using the risk ratio and 95% confidence interval (95% CI). Odds ratio (OR) or relative risk (RR) will be analyzed for dichotomous outcomes data, while the weighted mean difference (WMD) or the standard mean difference (SMD) will be analyzed for continuous outcomes.

#### 2.4.4 Dealing with missing data

If relevant data are missing in certain studies, we will manage to obtain missing data by contacting the authors of the corresponding studies via email. If the authors do not reply to our request, an intention-to-treat analysis will be employed in the absence of missing data.^[22]^

#### 2.4.5 Assessment of heterogeneity

Heterogeneity assessment will be quantified based on the Chi-squared test and *I*^2^ values, which are graded into 4 categories based on the Cochrane Handbook: little or no heterogeneity (*I*^2^: 0–40%); moderate heterogeneity (*I*^2^: 30–60%); substantial heterogeneity (*I*^2^: 50–90%); and considerable heterogeneity (*I*^2^: 75–100%).^[23]^

#### 2.4.6 Assessment for reporting biases

If there are sufficient studies included in meta analyses, reporting biases will be determined via funnel plots.

#### 2.4.7 Data synthesis and analysis

Data synthesis and analysis will be conducted by using Review Manager 5.3 (Cochran Collaboration, London, UK). Fixed effect model will be employed if little or no heterogeneity is detected; while random effect model will be used if considerable heterogeneity is detected (*I*^2^ ≥50%).

#### 2.4.8 Sensitivity analysis

To ascertain the robustness of meta-analysis results, we will conduct sensitivity analysis by removing certain RCTs during meta analyses in terms of different sample sizes, methodological quality of included studies, and other relevant factors.

#### 2.4.9 Subgroup analysis

Subgroup analysis will be conducted to explore the sources of heterogeneity when considerable heterogeneity is detected. If possible, subgroup analysis will be executed in terms of the following aspects: types of acupuncture (e.g., electro acupuncture, manual acupuncture, auricular acupuncture, and warming-needle moxibustion); types of control interventions; measurement time points of primary outcomes; and age groups of patients.

#### 2.4.10 Summary of evidence

The Grading of Recommendations Assessment, Development and Evaluation (GRADE)^[24]^ will be used to assess the strength of evidence for all meta-analysis outcomes, which is graded into 4 levels: high, moderate, low, and very low quality.

## 3 Discussion

VM is a recurrent vestibular disease that gains increasing attention by researchers and clinicians in recent years. It is characterized by vestibular symptoms and migrainous symptoms^[1]^ and has a significantly negative impact on patients’ daily life and work. Meanwhile, it will impair their psychology and/or emotional states. However, the pathophysiology of VM is poorly understood, and the majority of available hypotheses are based on the pathogenesis of migraine.

As conventional treatment, western medicine often just targets at single symptom of VM. Moreover, it is often limited by poor patient adherence because of its unavoidable side effects, such as nausea, dry mouth, and dizziness.^[25]^ As a major part in complementary and alternative medicine, acupuncture is frequently used for treating migraine, vertigo, and emotional disorders for a long history, which are primary symptoms and comorbidity of VM. In past few years, increasing clinical trials indicated that acupuncture can be used for treating migraine, vertigo, and emotional disorders synchronously, so it is likely to be an effective therapy for VM. There are also some published studies of RCTs in favor of acupuncture for treating VM.^[26,27]^ In addition, acupuncture has other advantages of simple operations, low medical cost, and less side effects. Nevertheless, to date, this is a lack of available evidence for the efficacy and safety of acupuncture for prophylaxis treatment of VM.

This present study will be the first systematic review and meta-analysis that aims to investigate the evidence of acupuncture for VM prophylaxis. It will verify whether acupuncture for VM prophylaxis is safe and effective. The findings of this study are expected to provide an alternative treatment option for VM patients and provide additional guideline in VM treatment for both clinicians and policy makers.

Nevertheless, the present study also has several limitations to be addressed. First, VM diagnostic criteria are relatively subjective, and there are no objective measurement indicators, which will have an impact on the inclusion criteria of patients. Second, given that the literature retrieved in this study will be restricted to English and Chinese mainstream databases, publication bias might exist. Third, at present, there may not be many eligible trials involving acupuncture for prophylaxis treatment of VM to be included in the meta-analysis.

## Author contributions

TYH and JH designed the trial protocol. TYH and AJZ drafted the manuscript. BJ and FFS planned a data analysis solution. All the authors have read, revised, and approved this version of the manuscript.

**Investigation:** Fengfei Shen.

**Writing – original draft:** Tianye Hu, Jin Hu.

**Writing – review & editing:** Aijun Zhang, Bin Jiang.

## Correction

The funding details have been updated to add “the Project of Zhejiang Administration of Traditional Chinese Medicine (grant no.2021ZQ084)”.
